# HOODS: finding context-specific neighborhoods of proteins, chemicals and diseases

**DOI:** 10.7717/peerj.1057

**Published:** 2015-06-30

**Authors:** Albert Palleja, Lars J. Jensen

**Affiliations:** 1 The Novo Nordisk Foundation Center for Protein Research, Faculty of Health and Medical Sciences, University of Copenhagen, Copenhagen N, Denmark; 2 The Novo Nordisk Foundation Center for Basic Metabolic Research, Faculty of Health and Medical Sciences, University of Copenhagen, Copenhagen Ø, Denmark

**Keywords:** Neighborhoods, Small-molecule compounds, Context-specific groups, Protein interaction network, Kinase inhibitors, Algorithm, Disease network

## Abstract

Clustering algorithms are often used to find groups relevant in a specific context; however, they are not informed about this context. We present a simple algorithm, HOODS, which identifies context-specific neighborhoods of entities from a similarity matrix and a list of entities specifying the context. We illustrate its applicability by finding disease-specific neighborhoods of functionally associated proteins, kinase-specific neighborhoods of structurally similar inhibitors, and physiological-system-specific neighborhoods of interconnected diseases. HOODS can be used via a simple interface at http://hoods.jensenlab.org, from where the source code can also be downloaded.

## Introduction

Clustering techniques have been used for decades to divide data into groups of similar entities. In molecular biology, algorithms such as hierarchical clustering, *k*-means, Markov clustering (MCL) and self-organizing maps have been extensively used to group genes with similar expression patterns (Eisen et al., 1998), to classify medical samples (Alizadeh et al., 2000), to construct protein families (Enright, Van Dongen & Ouzounis, 2002) and to cluster proteins into complexes based on protein interaction data (Krogan et al., 2006). Many authors have used clustered protein networks as a basis onto which they mapped other biological data to discover functional modules and protein complexes (Gavin et al., 2006). The clusters produced by clustering algorithms depend on the chosen parameters, e.g., *k* in *k*-means or Inflation in MCL. Yet, for a given biological problem there may be no choice of parameters that simultaneously produces all the desired clusters. The main reason for that is that these algorithms are not informed about the biological context of interest.

To produce context-specific groups of similar entities (neighborhoods), we have developed HOODS. Although it is similar to clustering methods in that it groups similar entities, it is not a clustering algorithm. The goal of clustering is to group entities together so that entities within a cluster are more related to each other than they are to entities in other clusters. By contrast, neighborhoods are defined in part by the similarity of the entities and in part by their association to the given context.

In the present paper, we present our method and show that it produces meaningful disease-specific neighborhoods when applied to a network of functionally associated proteins. We tested the performance of our algorithm on this task only to prove that it produces neighborhoods that are significantly better than random; it is not intended to compete with the many existing tools specifically designed for prioritizing disease genes (reviewed in Moreau & Tranchevent, 2012). Our method can be used in any case where the biomedical researcher needs to group entities based on a similarity network given a context. The context can optionally be defined as a weighted context with a score specifying how strongly each entity (e.g., a protein) is associated with the context (e.g., a disease). HOODs is designed to find additional entities from the entities already associated with the context by producing a set of context-specific network neighborhoods. The algorithm is not intended to filter the interactions of protein–protein interaction network that are related to a given context, a problem already addressed by others (Chowdhary et al., 2012; Schaefer et al., 2013). The filtering step of these tools may remove the additional context-specific entities that we want to find with our method. To illustrate the applicability of HOODS, we apply it to two additional, very different biomedical problems: finding kinase-specific neighborhoods of structurally similar inhibitors, and finding disease neighborhoods that relate to specific physiological systems. These examples were chosen because they make use of very different similarity metrics and context scores.

## Materials and Methods

### A simple algorithm to produce context-specific neighborhoods

**Figure 1:**
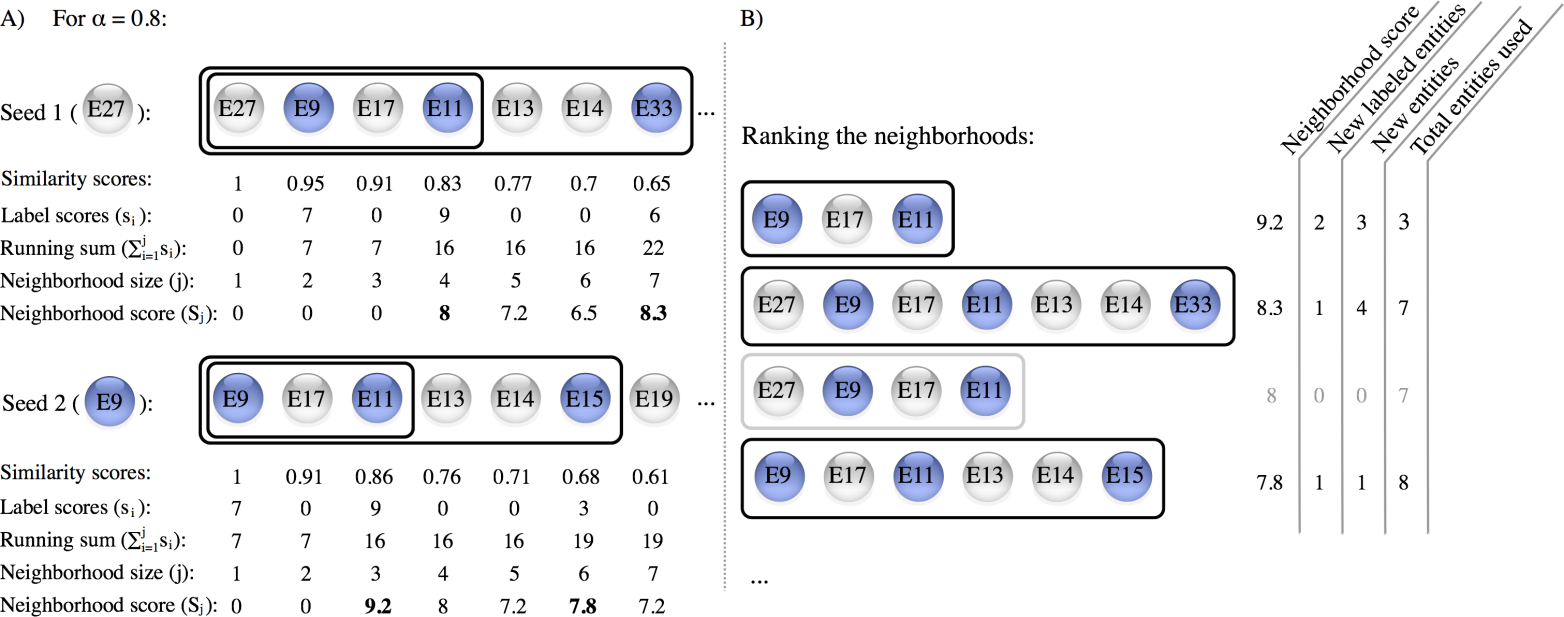
Outline of the algorithm. The example illustrates how HOODs calculates the neighborhood scores for each entity and selects a non-redundant subset of neighborhoods (A) For two seeds namely E27 and E9, we show the nearest neighbors sorted by similarity scores. Blue nodes indicate the labeled entities. Looping through the sorted nearest neighbors, the neighborhood score (using the default *alpha* value of 0.8) varies as both the running sum of the label scores and the neighborhood size change. Notice that the neighborhood score is only computed when the neighborhood contains at least two entities with a label score bigger than 0. The boxes delineate a new possible neighborhood each time a new labeled entity is encountered (these neighborhoods have their score in bold). (B) The algorithm next ranks the neighborhoods by score. To discard redundant neighborhoods, the algorithm loops over the ranked neighborhoods and counts: (i) the number of labeled entities not yet seen in higher ranked neighborhoods (“New labeled entities”), (ii) the number of entities not yet seen in higher ranked neighborhoods (“New entities”), and (iii) the total number of entities from the similarity matrix that have been used to build the set of neighborhoods (“Total entities used”). For instance, one of the neighborhoods obtained using E27 as seed is discarded because it adds no new labeled entities (box and numbers shown in gray).

HOODS identifies groups of related entities starting from two inputs: (1) a similarity *matrix* of the entities; and (2) a set of scores that associate entities with the context of interest (*s_i_*). The algorithm considers every entity in the *matrix* as a possible seed for a neighborhood. For each seed it goes through the nearest neighbors sorted by similarity and calculates a neighborhood score (*S_j_*) at every step (Fig. 1A). This score is defined as follows:

\begin{eqnarray*} {S}_{j}={\left(\displaystyle \sum _{i=1}^{j}{s}_{i}/j\right)}^{\alpha }\times {\left(\displaystyle \sum _{i=1}^{j}{s}_{i}\right)}^{1-\alpha }=\left(\displaystyle \sum _{i=1}^{j}{s}_{i}\right)/{j}^{\alpha }. \end{eqnarray*}

The parameter *α* is needed to set how much the user wants to penalize big neighborhoods and it ranges from 0 to 1. In this equation we take into account the label score density in our neighborhoods $\left(\sum _{i=1}^{j}{s}_{i}/j\right)$ and the total label score of them $\left(\sum _{i=1}^{j}{s}_{i}\right)$. The score is a weighted geometric average from these two components; ideally a neighborhood should have both a high density of labels and a large number of labeled entities.

The algorithm sorts all neighborhoods obtained from all seeds by the neighborhood score and filters out neighborhoods that contain the same set of labeled entities as a higher scoring neighborhood. It also removes neighborhoods that contain no entities that are not already included in any of the higher scoring neighborhoods (Fig. 1B).

### Disease protein network analysis

A global network of known and predicted interactions among human proteins was downloaded from the STRING database (Szklarczyk et al., 2011). Each interaction comes with an associated confidence score, which we used as the similarity *matrix*. Links between diseases and proteins were downloaded from the DISEASES database (Pletscher-Frankild et al., 2015), which we used as *labels*. These links are text-mined from literature and a *z*-score is calculated for each disease-protein association. Based on these associations, we created disease protein neighborhoods for each disease.

### Benchmarking

We used the disease-specific protein neighborhoods produced by HOODS to validate the method and to estimate a value for *α*. To these purposes, we performed leave-one-out cross-validation on a set of disease proteins from OMIM database (Amberger, Bocchini & Hamosh, 2011). To build a benchmark set consisting of actual disease proteins associated to diseases caused by disruptions in more than one protein, we chose 100 proteins encoded by single-gene loci and associated to 32 polygenic diseases in OMIM (Amberger, Bocchini & Hamosh, 2011). These disease associations from OMIM were only used for assessing the quality of the neighborhoods, whereas the text-mining derived labels described in the previous section were used to define the neighborhoods. To prove that our method is not biased by the fact that disease proteins are more studied than others, we repeated the leave-one-out cross-validation test choosing a random of the other 31 diseases as *labels*. We went through the top ranked neighborhoods again counting the proteins encountered before finding the left out proteins for the random disease label assigned, including all the proteins in the neighborhood containing them. To choose the random label, the only restriction was that both disease labels could not affect the same physiological system.

### Kinase inhibitor analysis

To analyze kinase inhibitors, we started from the activity screen of 178 compounds against 300 kinases published by Anastassiadis et al. (2011). We used the Tanimoto coefficients as similarity *matrix* among the inhibitors. These were calculated using Open Babel v2.2.3 with PF2 fingerprints (O’Boyle et al., 2011). We used as *labels* the percent inhibition caused by the compounds on a given kinase. Based on these we created compound neighborhoods for each of the resulting in 300 sets of labels.

### Disease network analysis

The disease network of Goh et al. (2007) is derived from OMIM. The interactions in the network represent shared genes, and we thus used the number of shared genes between each pair of diseases as the similarity *matrix*. The diseases had been classified into 22 categories based on which physiological system they affect. The label scores of all the diseases belonging to a category were set to 1 and used to produce a set of disease neighborhoods for each of the 22 physiological systems.

## Results and Discussion

### Validation of disease-specific protein neighborhoods produced by HOODS

**Figure 2:**
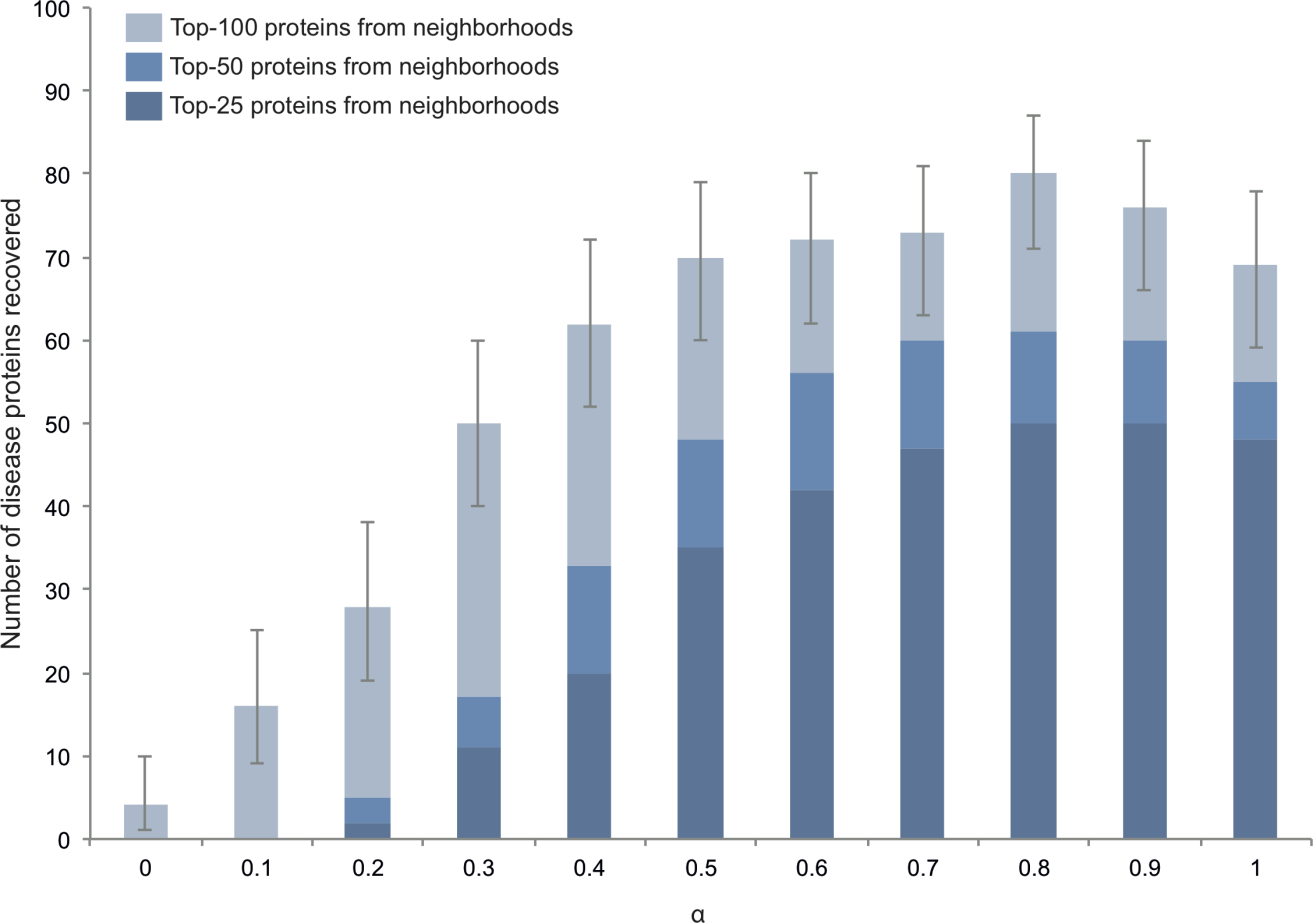
Validation of HOODS and estimation of *alpha* parameter. The bar chart shows the number of disease proteins correctly recovered before using 25, 50 or 100 proteins from the similarity matrix in the leave-one-out cross-validation of the method. The error bars represent the 95% confidence interval according to the Binomial distribution when using 100 proteins from the similarity matrix. For *alpha* values between 0.6 and 1, we observe similar performance, with 0.8 being the optimum.

We first used HOODS to identify disease-related protein neighborhoods using the human protein network from STRING (Szklarczyk et al., 2011) as *matrix* and text-mined disease–protein associations from DISEASES (Pletscher-Frankild et al., 2015) as *labels*. To validate our method and to estimate a value for *α* we performed leave-one-out cross-validation on a set of the 100 proteins encoded by single-gene loci associated to 32 polygenic diseases in OMIM (Amberger, Bocchini & Hamosh, 2011). Going through the ranked neighborhoods, we counted the total number of unique proteins encountered before finding the left out protein, including all the proteins in the neighborhood containing it (Fig. 1B). HOODS showed similar, good performance for *α* ranging from 0.6 to 1.0 (Fig. 2). We chose 0.8 as the default value for *α* because it is both the middle of this range and the value that gave the best performance, recovering 80 of the 100 proteins from the OMIM benchmark set among the first 100 proteins used to build the networks (Fig. 2). To show that the good performance is not purely due to disease proteins being more studied, we redid the leave-one-out cross-validation choosing a random of the other 31 diseases as *labels*. This recovered only 1 protein of the 100 proteins.

**Figure 3:**
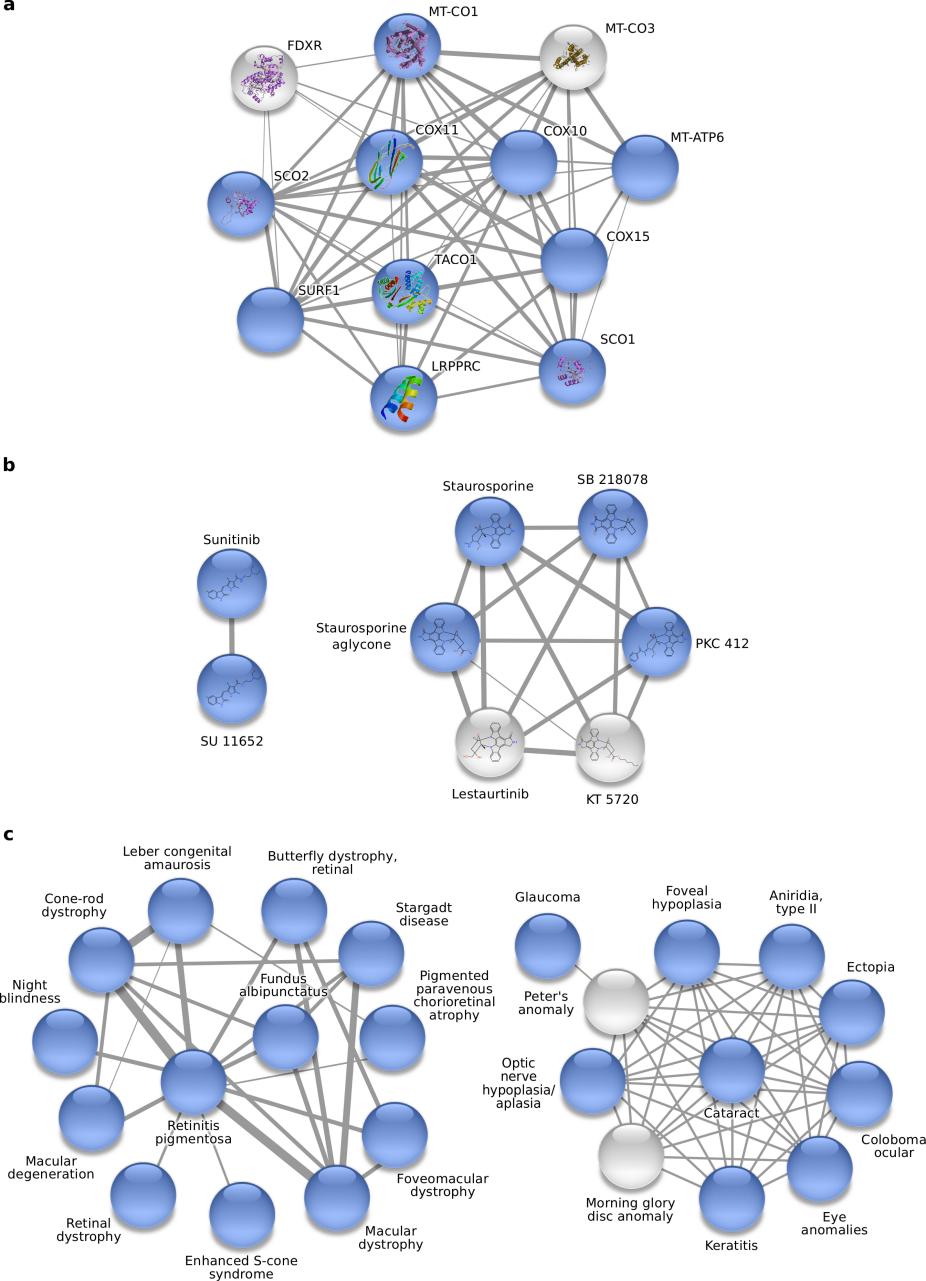
Example neighborhoods produced by HOODS. (A) A protein neighborhood related to Leigh disease. (B) Two neighborhoods of kinase inhibitors that target VEGFR2, and (C) Two disease neighborhoods of related eye diseases. In all examples, the labeled entities are shown in blue. For the disease protein neighborhood and the two kinase inhibitor neighborhoods, the molecular structures are shown within the nodes where available. The widths of the edges represent the relative similarities, which are based on the STRING interaction scores between the proteins, the Tanimoto coefficients between the chemical compounds, and the number of genes shared between the diseases, respectively.

As an example of the disease neighborhoods we chose the Leigh disease, which is a rare neurometabolic disorder caused by mutations in genes encoding subunits of the mitochondrial respiratory chain or assembly factors of respiratory chain complexes (Diaz et al., 2011). The highest scoring neighborhood with more than one protein not associated to the disease contains 12 proteins, 10 of which are labeled with the disease: 8 assembly factors of cytochrome c oxidase (COX) (Diaz et al., 2011); one mitochondrial COX subunits (Diaz et al., 2011); one mitochondrial ATP synthase subunit (Kucharczyk, Rak & di Rago, 2009). In addition, there are two proteins that were not labeled with the disease, namely MT-CO3, and FDXR (Fig. 3A). MT-CO3 is one of three mitochondrial COX subunits and has already been linked to Leigh disease (Tiranti et al., 2000); the text mining strategy failed to find this link, but HOODS was able to recover it. FDXR is a new candidate protein that is likely to be involved in Leigh disease considering their role in the biosynthesis and covalent attachment of the prostetic heme A group of COX (Barros & Tzagoloff, 2002; Moraes, Diaz & Barrientos, 2004; Diaz et al., 2011).

### Kinase-specific inhibitor neighborhoods

To test if HOODS can also be applied to chemoinformatics problems, we used it to group kinase inhibitor with similar chemical structure that target a given kinase. Figure 3B shows the results obtained using the inhibition data for the tyrosine kinase VEGFR2, the malfunction of which is related to angiogenesis-dependent cancers such as renal cell carcinoma (RCC) and gastrointestinal stromal tumor (GIST) (Demetri et al., 2006; Motzer et al., 2007). We obtained two neighborhoods of VEGFR2 inhibitors. The first comprises Sunitinib and the structurally similar compound SU 11652. Sunitinib targets multiple receptor tyrosine kinases including the VEGFR kinases and is used to treat RCC and imatinib-resistant GIST (Demetri et al., 2006; Motzer et al., 2007). The second neighborhood contains six compounds: Staurosporine, three Staurosporine derivates and two other compounds similar to Staurosporine (Lestaurtinib and KT 5720). The Staurosporine-like compounds are known for their high promiscuity, i.e., they inhibit many kinases.

### Physiological system-specific disease neighborhoods

Mutations in genes are the molecular causes of many human diseases. Because many genes are involved in multiple diseases, the network of human diseases is highly interconnected (Vidal, Cusick & Barabasi, 2011). We tested the ability of HOODS to identify neighborhoods of diseases affecting certain physiological systems within the disease network published by Goh et al. (2007). To illustrate the results, we show two of the highest scoring neighborhoods for ophthalmological diseases (Fig. 3C). The first contains 13 ophthalmological diseases. The second neighborhood contains 9 ophthalmological, one skeletal (morning glory disc anomaly) and one developmental disease (Peter’s anomaly). Although the latter two were not classified as ophthalmological diseases, they are both eye diseases affecting the optic nerve and the cornea, respectively.

### Usage of HOODS

To enable the usage of HOODS in an easy way without programming skills needed, we built a simple interface at http://hoods.jensenlab.org. This interface allows the users to upload their proteins of interest from human or budding yeast as *labels* and use the human or budding yeast protein interactions from STRING (Szklarczyk et al., 2011) as similarity *matrix*. However, the interface it is not restricted to work only with human and yeast proteins, the users can also upload their own *labels* and customized similarity *matrices* from other organisms or from other biomedical fields. For analysis of large datasets, we recommend that users download the program from the website and run it on their own computers.

## Conclusions

We have presented a new algorithm, HOODS, for identifying context-specific neighborhoods. The algorithm produces meaningful results for data from a variety of biomedical disciplines, which highlights its applicability within bioinformatics, chemoinformatics, and medical informatics alike. HOODS is available from http://hoods.jensenlab.org, where it can be either used through a simple web interface or downloaded for local installation.
